# Antioxidant Properties of Seeds from Lines of Artichoke, Cultivated Cardoon and Wild Cardoon

**DOI:** 10.3390/antiox2020052

**Published:** 2013-06-17

**Authors:** Alessandra Durazzo, Maria Stella Foddai, Andrea Temperini, Elena Azzini, Eugenia Venneria, Massimo Lucarini, Enrico Finotti, Gianluca Maiani, Paola Crinò, Francesco Saccardo, Giuseppe Maiani

**Affiliations:** 1 CRA-Ex INRAN (National Research Institute on Food and Nutrition), Via Ardeatina 546, Rome 00178, Italy; E-Mails: foddai@inran.it (M.S.F.); azzini@inran.it (E.A.); venneria@inran.it (E.V.); lucarini@inran.it (M.L.); finotti@inran.it (E.F.); gianluca.maiani@alice.it (G.M.); maiani@inran.it (G.M.); 2 Department of Science and Technologies for Agriculture, Forestry, Nature and Energy (DAFNE), Tuscia University, Via San Camillo De Lellis snc, Viterbo 01100, Italy; E-Mails: andreatemperini@libero.it (A.T.); migammabeta@yahoo.it (F.S.); 3 Technical Unit for Sustainable Development and Innovation of Agro-Industrial System (UTAGRI), ENEA, Casaccia C.R., Via Anguillarese 301, Rome 00123, Italy; E-Mail: paola.crino@enea.it

**Keywords:** *Cynara cardunculus*, seeds, aqueous-organic extract, residue, FRAP, TEAC

## Abstract

The artichoke (*Cynara cardunculus* L. subsp. *scolymus* L.), the cultivated cardoon (*Cynara cardunculus* var. *altilis* DC.) and the wild cardoon (*Cynara cardunculus* var. *sylvestris* L.) are species widely distributed in the Mediterranean area. The aim of this research was to evaluate the antioxidant properties of seeds from lines of artichoke and cultivated and wild cardoon in both aqueous-organic extracts and their residues by FRAP (Ferric Reducing Antioxidant Power) and TEAC (Trolox Equivalent Antioxidant Capacity) evaluations. Both artichoke and cardoon seeds are a good source of antioxidants. Among artichoke seeds, hydrolysable polyphenols contribution to antioxidant properties ranged from 41% to 78% for FRAP values and from 17% to 37% for TEAC values. No difference between cultivated and wild cardoon in antioxidant properties are reported. Our results could provide information about the potential industrial use and application of artichoke and/or cardoon seeds.

## 1. Introduction

The artichoke (*Cynara cardunculus* L. subsp. *scolymus* L.), the cultivated cardoon (*Cynara cardunculus* var. *altilis* DC.) and the wild cardoon (*Cynara cardunculus* var. *sylvestris* L.) are species widely distributed in the Mediterranean area. Wild cardoon is the ancestor of both the cultivated forms, which evolved separately as a result of different selection criteria [1,2].

These plants were popular in Greeks and Romans as food and medicine [3]. Nowadays, they are considered as functional foods, owing to their nutritional properties [4,5,6].

A lot of investigations on bioactive molecules (mainly represented by flavonoids such as apigenin and luteolin and by hydroxycinnamic derivatives such as mono- and di-caffeoylquinic acids) and antioxidant properties of edible part of artichoke and cardoon plant have been carried out [7,8,9,10,11], few studies on antioxidant properties of *Cynara* seeds are present in literature [12,13].

According to Foti *et al.* [14] the chemical composition of artichoke seeds was the following: crude protein 21.6%, crude fiber 17.1%, crude oil 24.05% and ash 3.8%. Raccuia and Melilli [15] have shown like *Cynara* oil is suitable for human consumption.

Saponins, sesquiterpene lactones, flavones, sterols, coumarins and lignans have been identified and quantified in seeds of *Cynara* [12,13]. Georgieva *et al.* [13], by comparing the radical scavenging capacity towards 1,1-diphenyl-2-picrylhydrazyl (DPPH) of ethanol extracts prepared from *Cynara scolymus* L. seeds and leaves, have demonstrated that at any studied concentration of the seeds extract the percent of the scavenged DPPH radicals was considerably higher than that calculated for the leaves extract.

Among the various kinds of natural antioxidants, polyphenols have received much attention. These compounds exhibit a wide range of physiological properties, such as anti-allergic, anti-atherogenic, anti-inflammatory, antimicrobial, antioxidant, anti-thrombotic, cardioprotective, and vasodilatory effects [16,17].

Polyphenols exist as easily extractable compounds (free) (compounds solubilized by aqueous organic solvents) and as less extractable types (bound) (compounds that remain in their corresponding extraction residues) [18,19,20,21,22,23,24,25].

Recent investigations have used an alkaline hydrolysis, acid hydrolysis, or enzymatic digestion [26,27,28,29,30]; polyphenols content of plant foods have been underestimated: significant amounts of bioactive compounds remain in the residue from extraction as non extractable polyphenols [31,32].

Arranz *et al.* [23] have concluded, by studying dietary polyphenols in cereals, fruits, vegetables, nuts, and legumes, that non extractable polyphenols are the major part of dietary polyphenols. Studies of non extractable polyphenols are few respect to those on extractable polyphenols [33,34]. No studies addressed to the estimation of antioxidant properties in aqueous-organic extracts and residues of *Cynara* seeds are present in literature.

In this work extraction and isolation of aqueous-organic extracts of foods (extractable polyphenols) and residues (non-extractable polyphenols) of seeds from lines of artichoke and cultivated and wild cardoon were optimized.

The aim of this research was to evaluate the antioxidant properties of seeds from lines of artichoke, cultivated and wild cardoon and hybrids by FRAP (Ferric Reducing Antioxidant Power) and TEAC (Trolox Equivalent Antioxidant Capacity) evaluations.

The distinction between free and bond antioxidants represents a key element to define the potential of seeds. Thus, a recent increase in serious research on the commercial application of antioxidants, led to the additional focus on the topical relevance of food derived extracts in the present study [35,36,37].

## 2. Experimental Section

### 2.1. Chemicals and Standards

All solvents were of HPLC or optima grade and purchased from Carlo Erba (Milan, Italy). Common reagents and standards were purchased from Sigma–Aldrich srl (Milan, Italy) and were of the highest available grade. Double-distilled water (Millipore, Milan, Italy) was used throughout the study.

### 2.2. Sample Preparation

The seeds were ground in a refrigerated mill (Janke and Kunkec Ika Labortechnik, Germany) and the pulverized samples were sieved to obtain a granulometry of 0.5 mm. The descriptions of selected seeds are reported in Table 1.

**Table 1:** Description of selected seeds.

Species	Lines	Abbreviation
Artichoke-male fertile lines		
	Brawley North	BN
	Cavi	CA
	Cyl	CYL
	F19	F-19
	Vert de Provence	VP
	White bloomer	WB
	22BD	22BD
Cultivated Cardoon		
	Bianco avorio	BA
	Belgio	BE
	F.S.	F.S.
	Madrid	MA
Wild Cardoon		
	Cerveteri	CE
	Siena	S
	Tarquinia	T
Cardoon/Artichoke Hybrides		
F1 hybrid	Madrigal	MG
F1 hybrid	Romolo	RO
cardoon × artichoke hybrid	F1 103	F1 103
cardoon × artichoke hybrid	F1 104	F1 104
F1 hybrid (synthetic variety)	Istar	I

### 2.3. Extraction for Evaluation of Antioxidant Properties

Total phenolic compounds were extracted as described by Durazzo *et al.* [38] with minor modifications as follows. Aqueous-organic extracts (extractable polyphenols) and their residues (non-extractable polyphenols) were isolated and studied. In particular in residues, among non-extractable polyphenols, hydrolysable polyphenols (comprising hydrolysable tannins, phenolic acids and hydroxycinnamic acids that are released from the food matrix by strong acidic hydrolysis) were determined.

*Aqueous-organic extract*. About 3–4 g of samples were placed in a test tube and 20 mL of acidic methanol/water (50:50 v/v, pH 2) were added. The tubes were vortexed at room temperature for 3 min, followed by 1 h shaking in a water bath at room temperature. The tube was centrifuged at 2500 *g* for 10 min, and the supernatant was recovered. Twenty milliliters of acetone/water (70:30, v/v) were added to residue, then vortexing, shaking and centrifugation were repeated. Both methanolic and acetonic extracts were combined and centrifuged at 3500 *g* for 15 min. The resulting supernatant was transferred into tubes and directly used for the determination of antioxidant capacity.

*Residue*. Residues were left in a ventilating and heating apparatus (max temperature 25 °C), until dryness. Briefly, 300–400 mg of the residue were mixed with 20 mL of methanol and 2 mL of concentrated sulfuric acid (18 M). The samples were gently stirred for 1 min and were shaken in a water bath at 85 °C for 20 h.

The samples were then centrifuged (3000 *g* for 10 min), and the supernatant was recovered. After two washings with minimum volumes of distilled water and recentrifuging as necessary, the final volume was taken up to 50 mL. The tube was centrifugated at 3500 *g* for 20 min and was transferred into tubes and directly used for the determination of antioxidant capacity.

### 2.4. Antioxidant Properties Determination

Antioxidant properties have been determined in both aqueous-organic extracts and their residues using two different assays, Ferric Reducing-Antioxidant Power (FRAP) and Trolox Equivalent Antioxidant Capacity (TEAC).

The FRAP method, according to Benzie and Strain [39] and Pulido *et al.* [40], is based on the reduction of Fe^3+^-TPTZ (2,4,6-tripyridyl-s-triazine) complex to ferrous at low pH through the use of a Tecan Sunrise^®^ plate reader spectrophotometer. The TEAC assay measures the ability of antioxidants to quench radical cations according to Re *et al.* [41].

### 2.5. Statistical Analysis

All analyses were performed in triplicate. Data are presented as mean ± Standard Deviation (SD). Statistica for Windows statistical package (release 4.5; StatSoft Inc., Vigonza PD, Italy) was used to perform One-Way Analysis of Variance (ANOVA).

## 3. Results and Discussion

The chemical diversity of natural phenols makes it difficult to separate, detect, and quantify these molecules from a complex food matrix. For determination of antioxidant capacity of foods, it is important to consider three steps: extraction of antioxidants, antioxidant capacity measurements and expression of results [42,43,44]. It is generally known that chemical extraction depends on type of solvents, extraction time, and temperature as well as on the chemical compositions and physical characteristics of the sample [44,45].

In Table 2 FRAP values (mmol/kg d.w.) and TEAC values (mmol/kg d.w.) of aqueous-organic extracts (combining two extraction cycles) and the corresponding residues of selected seeds were reported.

For artichoke seeds, FRAP values are placed within the range 248.3–344.9 mmol/kg d.w. in aqueous-organic extracts and enlarged range of 185.1–1067.2 mmol/kg d.w. in residues. The type White Bloomer (WB) reached the highest FRAP values both in aqueous-organic extract and residue; TEAC values ranged from 63.3 ± 3.2 to 106.3 ± 7.7 mmol/kg d.w. in aqueous-organic extracts and from 21.5 ± 1.6 to 45.6 ± 7.1 mmol/kg d.w. In our work the hydrolysable polyphenols contribution to antioxidant properties ranged from 41% to 78% for FRAP values and from 17% to 37% for TEAC values; the genotype CYL exhibited the highest contribution both for FRAP and TEAC values. It is interesting to notice that in most of artichoke seeds the hydrolyzable polyphenols presented higher ferric reducing powers than extractable polyphenols. As reported in literature data, non extractable polyphenols are more abundant than extractable polyphenols in many foodstuffs [32]. High antioxidant capacity of hydrolysable phenolics was found in the residues of aqueous-organic extracts in cereals [19] and walnuts [46]. Saura-Calixto *et al.* [47], studying total polyphenol content of plant foods in the Spanish diet, reported that hydrolysable polyphenols were a quantitatively important fraction of polyphenols in all food groups.

For cardoons, FRAP values ranged from 255.4 ± 22.4 to 358.8 ± 18.30 mmol/kg d.w. for aqueous-organic extracts and from 206.6 ± 80.1 to 733.8 ± 56.4 mmol/kg d.w for residues, whereas TEAC values ranged from 75.3 ± 5.1 to 119.0 ± 1.3 mmol/kg d.w. for aqueous-organic extracts and from 19.1 ± 3.1 to 42.0 ± 2.2 mmol/kg d.w.for residues; no difference in antioxidant properties between cultivated and wild cardoon are reported. Among cultivated cardoons, in both fractions the lowest FRAP and TEAC values were found for type Madrid (MA). Among wild forms, the genotype Siena (S) reached the highest TEAC values in both fractions and the highest FRAP values in residues, and this genotype exhibited the hydrolysable polyphenols contributions of 68% for FRAP values and of 31% for TEAC values.

Among hybrids, FRAP values ranged from 231.3 ± 18.0 to 331.8 ± 12.3 mmol/kg d.w. in aqueous-organic extracts and from 159.8 ± 39.4 to 391.5 ± 30.1 mmol/kg d.w. in residues; the hydrolysable polyphenols contribution to antioxidant properties ranged from 37% to 61% for FRAP values and from 13% to 25% for TEAC values.

**Table 2:** Ferric Reducing Antioxidant Power (FRAP) values (mmol/kg d.w.) and Trolox Equivalent Antioxidant Capacity (TEAC) values (mmol/kg d.w.) of selected seeds *.

Crop	FRAP		TEAC	
	Aqueous-organic extract	Residue	Aqueous-organic extract	Residue
*Artichoke*				
BN	278.5 ± 18.8 ^ab^	249.3 ± 51.0 ^a^	71.4 ± 3.8 ^a^	31.5 ± 11.7 ^ab^
CA	262.3 ± 17.0 ^a^	185.1 ± 6.6 ^a^	106.3 ± 7.7 ^c^	21.5 ± 1.6 ^a^
CYL	248.3 ± 16.3 ^a^	898.4 ± 65.4 ^b^	63.3 ± 3.2 ^a^	36.7 ± 6.2 ^ab^
F-19	310.2 ± 28.7 ^b^	333.7 ± 69.3 ^a^	87.9 ± 5.6 ^b^	25.4 ± 5.6 ^a^
VP	344.9 ± 17.9 ^c^	616.3 ± 39.4 ^c^	87.4 ± 9.6 ^b^	45.6 ± 7.1 ^b^
WB	320.0 ± 25.2 ^bc^	1067.2 ± 173.1 ^d^	72.85 ± 2.5 ^a^	36.6 ± 2.7 ^ab^
22BD	273.6 ± 7.2 ^ab^	704.3 ± 57.0 ^c^	64.3 ± 2.1 ^a^	34.4 ± 8.1 ^ab^
*Cultivated Cardoon*				
BA	352.3 ± 22.2 ^b^	708.9 ± 73.4 ^b^	96.2 ± 4.1 ^b^	30.0 ± 1.8 ^ab^
BE	326.1 ± 34.1 ^b^	298.6 ± 76.2 ^a^	104.4 ± 7.0 ^b^	38.7 ± 14.9 ^b^
F.S.	333.1 ± 23.0 ^b^	206.6 ± 80.1 ^a^	119.0 ± 1.3 ^c^	19.1 ± 3.1 ^a^
MA	255.4 ± 22.4 ^a^	265.4 ± 98.6 ^a^	85.8 ± 2.0 ^a^	29.7 ± 11.0 ^ab^
*Wild Cardoon*				
CE	340.9 ± 24.2	301.3 ± 20.0 ^a^	78.2 ± 1.7 ^a^	23.1 ± 0.8 ^a^
S	343.2 ± 8.8	733.8 ± 56.4 ^c^	93.6 ± 9.0 ^b^	42.0 ± 2.2 ^c^
T	358.9 ± 18.3	629.2 ± 52.7 ^b^	75.3 ± 5.1 ^a^	28.7 ± 1.8 ^b^
*Cardoon/Artichoke Hybrides*				
MG	231.3 ± 18.0 ^a^	159.8 ± 39.4 ^a^	95.8 ± 3.6 ^a^	18.6 ± 1.9 ^a^
RO	252.7 ± 20.6 ^ac^	391.5 ± 30.1 ^b^	94.9 ± 2.5 ^a^	32.1 ± 2.0 ^c^
F1 103	292.2 ± 22.6 ^bc^	219.9 ± 33.8 ^a^	110.7 ± 2.4 ^b^	19.6 ± 0.6 ^a^
F1 104	280.2 ± 10.5 ^c^	166.5 ± 30.9 ^a^	123.5 ± 2.6 ^c^	17.9 ± 3.3 ^a^
I	331.8 ± 12.3 ^d^	238.0 ± 47.2 ^a^	122.3 ± 7.2 ^c^	24.5 ± 0.7 ^b^

* mean ± S.D.; a–d: Anova, Tukey HSD Test: within samples of each type of seeds, by columns, means followed by different letters are significantly different (*p* < 0.05).

Among hybrids, FRAP values for aqueous-organic extracts can be placed in the following decreasing order of concentration: I > F1 103 > F1 104 > RO > MG, whereas the type Romolo (RO) reached the highest FRAP values in residue; this type reached the highest TEAC values in residue too, exhibiting the highest contribution of hydrolysable polyphenols to antioxidant properties both for FRAP and TEAC values.

## 4. Conclusions

Both artichoke and cardoon seeds represent a rich source of antioxidants. These seeds could represent a good source of health-promoting bioactive compounds and therefore encourage a nutraceutical use of this species. Among artichoke seeds, hydrolysable polyphenols contribution to antioxidant properties ranged from 41% to 78% for FRAP values and from 17% to 37% for TEAC values. No difference between cultivated and wild cardoon in antioxidant properties are reported.

The results of this study contribute to the better knowledge of biochemical composition of the artichoke/cardoon seeds. The high antioxidant content of seeds makes this vegetable a potential useful source in nutritional as well as industrial field (agro-industrial applications).
